# Prognostic value of *PNN* in prostate cancer and its correlation with therapeutic significance

**DOI:** 10.3389/fgene.2022.1056224

**Published:** 2022-11-16

**Authors:** Ruisong Wang, Ziyi Qin, Huiling Luo, Meisen Pan, Mingyao Liu, Pinhong Yang, Tieliu Shi

**Affiliations:** ^1^ College of Life and Environmental Sciences, Hunan University of Arts and Science, Changde, Hunan, China; ^2^ Changde Research Centre for Artificial Intelligence and Biomedicine, Changde, China; ^3^ Affiliated Hospital of Hunan University of Arts and Science (the Maternal and Child Health Hospital), Changde, Hunan, China; ^4^ Medical College, Hunan University of Arts and Science, Changde, Hunan, China; ^5^ Hunan Provincial Ley Laboratory for Molecular Immunity Techonology of Aquatic Animal Diseases, Changde, China

**Keywords:** prognosis signature, *PNN*, immune infiltration, drug prediction, methylation status, prostate cancer

## Abstract

Prostate cancer (PCa) is the most common malignancy. New biomarkers are in demand to facilitate the management. The role of the pinin protein (encoded by *PNN* gene) in PCa has not been thoroughly explored yet. Using The Cancer Genome Atlas (TCGA-PCa) dataset validated with Gene Expression Omnibus (GEO) and protein expression data retrieved from the Human Protein Atlas, the prognostic and diagnostic values of *PNN* were studied. Highly co-expressed genes with PNN (HCEG) were constructed for pathway enrichment analysis and drug prediction. A prognostic signature based on methylation status using HCEG was constructed. Gene set enrichment analysis (GSEA) and the TISIDB database were utilised to analyse the associations between *PNN* and tumour-infiltrating immune cells. The upregulated *PNN* expression in PCa at both transcription and protein levels suggests its potential as an independent prognostic factor of PCa. Analyses of the *PNN*’s co-expression network indicated that *PNN* plays a role in RNA splicing and spliceosomes. The prognostic methylation signature demonstrated good performance for progression-free survival. Finally, our results showed that the *PNN* gene was involved in splicing-related pathways in PCa and identified as a potential biomarker for PCa.

## Introduction

Prostate Cancer (PCa) is the third most common cancer overall (Pan et al., 2017) and the most common malignant tumour in the male genitourinary system (Ren et al., 2017; Caggiano et al., 2019; Jambor et al., 2019). Its prevalence and mortality vary greatly depending on race and geographic location (Lindberg et al., 2013). At present, PCa is usually screened and diagnosed through digital rectal examination (DRE), prostate-specific antigen (PSA) value, Gleason score by prostate biopsy, and magnetic resonance imaging (MRI) of the prostate (Patil and Gaitonde, 2016). New biomarkers used with techniques such as liquid biopsy and imaging have also been used for clinical diagnosis (Kim et al., 2016; Li et al., 2018; Law et al., 2020). In fact, metastatic PCa remains incurable despite promising advances in biomedical research. Therefore, patients’ good prognosis is currently dependent on early detection. Conventional non-surgical options for PCa therapy include androgen deprivation therapy (ADT), radiotherapy (RT), ablation therapy, chemotherapy, and emerging immunotherapy. However, the effectiveness of the drugs including abiraterone and enzalutamide, are limited and temporary, but has been established clinically.

New biomarkers for diagnosis and treatment need to explore the mechanism deeply. In the past two decades, several mechanisms of PCa have been continuously reported, including novel associations of androgen signalling (Caggiano et al., 2019; Cioni et al., 2020), *TP53* signalling (Ecke et al., 2010; Liu et al., 2021), and the Wnt signalling pathway (Murillo-Garzón et al., 2018; Datta et al., 2020) with the disease. In fact, it is now believed that various cytokines and intercellular signals regulate PCa during its development (Cucchiara et al., 2017). Thus, many potential mechanisms of PCa remain to be explored, which may lead to new diagnostic techniques or therapeutic strategies, especially for metastatic PCa.

The pinin protein, reported as a desmosome-associated protein encoded by the *PNN* gene, is a phosphoprotein rich in serine and arginine with a molecular size of 140 kDa. Recently, it has been suggested that pinin is associated with cell adhesion (Tang et al., 2020; Yao and Ma, 2020). It serves as a putative tumour promoter by reversing the expression of E-cadherin (Simon et al., 2015). The upregulation of pininhas been reported to enhance metastasis in colorectal cancer (Wei et al., 2016), triple-negative breast cancer cells (Kang et al., 2020), pancreatic cancer (Yao and Ma, 2020), and nasopharyngeal carcinoma cells (Tang et al., 2020). As an oncogenic factor, *PNN* can protect hepatocellular carcinoma cells from apoptosis (Yang et al., 2016) and promote cell adhesion in ovarian cancer (Zhang et al., 2016), as well as renal cell carcinoma (Jin et al., 2021). These studies indicate the critical role of *PNN* in metastasis; thus, it could be a potential biomarker for some tumours. However, the role of pininin PCa progression has not been thoroughly studied yet. Since the tumour microenvironment (TME) has emerged as a critical factor in metastasis (Yin et al., 2019; Yuan et al., 2022), there may also be a functional linkage between TME and *PNN* in PCa, but this hypothesis remains to be investigated.

Since the *PNN* gene has not been comprehensively deciphered in PCa, we conducted a series of studies on its roles in patients’ survival and prognosis, as well as in immune infiltration in PCa through various bioinformatic approaches. We explored the expression pattern of the *PNN* gene and its potential prognostic value for PCa. We also investigated the relationship between *PNN* and the tumour immune microenvironment (TIME), which could facilitate understanding the mechanism of immunotherapy for PCa and lead to the discovery of a prognosis signature or novel therapeutic targets.

## Materials and methods

**FIGURE 1:**
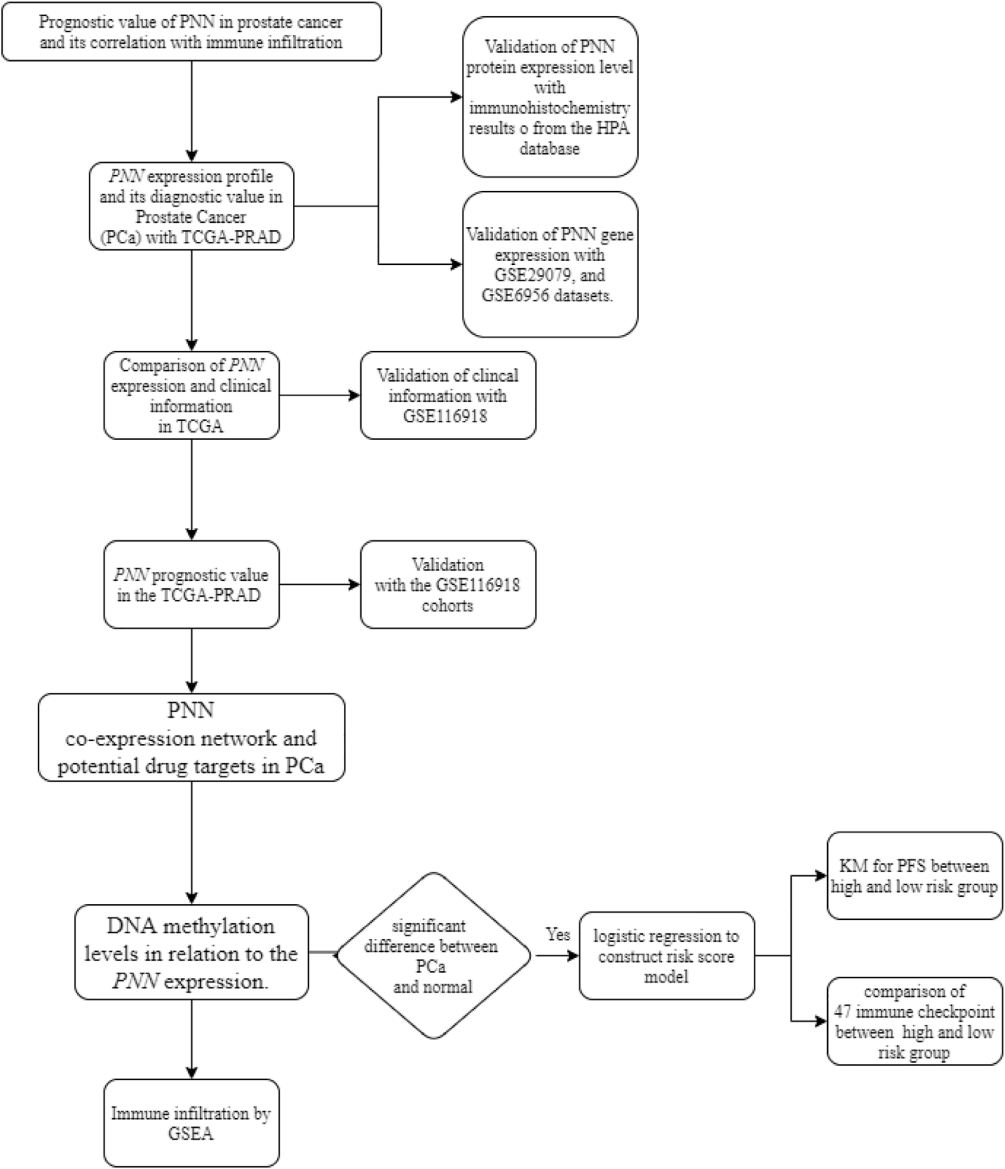
Analysis pipeline of *PNN* performed in this study.

To illustrate the function of *PNN* in PCa, we conducted a comprehensive bioinformatic analysis using multiple datasets. The whole analysis pipeline performed here is displayed in Figure 1.

### Data source

The transcriptome data [the level 3 mRNA expression data (FPKM), normalized using $\log_{2}\left(FPKM+1\right)$ ] of normal tissues (52 cases) and tumour tissues with complete clinical information (379 cases) were extracted from The Cancer Genome Atlas (TCGA) database of prostate adenocarcinoma (PRAD). The mRNA expression profiles contained in the GSE116918 (Jain et al., 2018), GSE29079 (Börno et al., 2012), and GSE6956 (Wallace et al., 2008) datasets, which were normalized by their corresponding providers, were downloaded from Gene Expression Omnibus (GEO) database. A total of 248 PCa cancer samples with clinical information were included in the GSE116918 dataset. The GSE29079 dataset contained 48 normal samples and 47 PCa samples, while the GSE6956 dataset had 18 normal samples and 69 PCa samples. However, neither GSE29079 nor GSE6956 contains clinical information. The BioGRID database offered 253 unique interactors of pinin with experimental pieces of evidence (Oughtred et al., 2021). TSVdb offered *PNN* splicing variants expression (Sun et al., 2018). For *PNN* expression in pan-cancer, we downloaded the standardised pan-cancer dataset TCGA TARGET GTEx (PANCAN, N = 19131, G = 60499) from the UCSC (https://xenabrowser.net/) database and further extracted the expression data of *PNN* gene in each sample. In addition, we filtered out the samples with zero expression levels, and further transformed each expression value with log2 (x + 0.001), finally, we excluded those with less than three samples in a single cancer species.

### Protein expression analysis with the Human Protein Atlas database

The Human Protein Atlas (HPA) provides the protein expression of pinin in normal prostate (*via* https://www.proteinatlas.org/ENSG00000100941-PNN/tissue/prostate) and tumour tissues (*via* https://www.proteinatlas.org/ENSG00000100941-PNN/pathology/prostate+cancer) (Uhlén et al., 2015). All images of tissues in HPA database are stained by immunohistochemistry. We extracted the immunohistochemistry images directly from the HPA database.

### Independent prognostic analysis

Correlation analysis of *PNN* expression and clinicopathological characteristics was performed. The expression of *PNN* between the subgroups was compared based on the following clinicopathological features: age (<60 or ≥60 years old), N stage (N0, N1), M stage (M0, M1), T stage (T2, T3, T4), surgical margin (R0, R1, R2, RX), prostate-specific antigen (PSA) level (<10 or ≥10 years), and Gleason score (6, 7, 8, 9, 10). Univariate and multivariate Cox regression analyses were implemented to identify independent predictors of survival in the TCGA-PRAD and GSE116918 datasets.

### Expression profiles of *PNN* gene in primary and metastatic prostate cancer

We downloaded GSE38241 (Aryee et al., 2013) and GSE25136 (Sun and Goodison, 2009) datasets (the authors processed normalisation) from GEO. For the merging of these datasets, we used the method of COMBAT (Johnson et al., 2007), implemented in the R package inSilicoMerging (Taminau et al., 2012) to obtain the expression matrix. Finally, the *PNN* expression was compared using the Kruskal-Wallis test.

### Construction of the *PNN* co-expression network

We calculated the Pearson correlation of all genes (RNA-seq) in the TCGA dataset with *PNN* using the Linkomics database (http://www.linkedomics.org/) and selected the genes with correlation coefficients > 0.8 and *p* < 0.05 as *PNN* co-expressed genes.

### Functional and pathway enrichment analysis

The “clusterProfiler” R package was utilised to conduct Gene Ontology (GO) and Kyoto Encyclopedia of Genes and Genomes (KEGG) analysis (Yu et al., 2012). GO enrichment analysis mainly described the biological processes (BP), cellular components (CC), and molecular functions (MF) correlated with genes. The threshold for significant enrichment was set as a *p*-value < 0.05 or FDR < 0.05, as stated. Single sample gene set enrichment analysis (ssGSEA) enrichment scores were calculated in each sample using the “GSVA” package of R (Hänzelmann et al., 2013).

### Identification of potential drugs

In this research, potential drug (or molecules) was predicted using the Drug Signatures database (DSigDB) *via* Enrichr (https://maayanlab.cloud/Enrichr/) based on the *PNN* gene as well as the positively co-expressed gene with *PNN* (correlation coefficient > 0.8 and *p* < 0.05) (Chen et al., 2013; Kuleshov et al., 2016; Xie et al., 2021).

### DNA methylation analysis and construction of the prognostic signature

The CpG sites in the promoter of *PNN* and *PNN*’s co-expressed genes were obtained from the MEXPRESS database (Koch et al., 2015; Koch et al., 2019). A univariate Cox analysis in R was used to determine the association between methylation levels at each CpG site and progression-free survival (PFS) for each patient, and *p* < 0.01 was considered statistically significant. Candidate prognostic CpG sites were selected using the Least Absolute Shrinkage and Selection Operator (LASSO) algorithm. Based on the candidate CpG sites generated from the above algorithm, a multivariate Cox regression model was used to construct a prognostic signature. The RiskScore of each recipient was calculated using the following formula:

$$RiskScore=\Sigma_{i=1}^{n}\;\beta i\times {Meth}_{i}$$

In which β refers to coefficient, and Meth refers to the level of methylation.

Patients were divided into the high-risk ( RiskScore≥median ) and low-risk groups ( RiskScore<median ) in the TCGA dataset. Then, we performed ROC analysis using the R software package pROC (version 1.17.0.1) to obtain the AUC. The R package “survival” was used to perform the two risk groups’ Kaplan-Meier (KM) survival analysis.

### Gene set enrichment analysis

To inspect the different signalling pathways between the *PNN* low- and high-expression groups in the TCGA-PRAD dataset, Gene Set Enrichment Analysis (GSEA) was conducted by the “clusterProfiler” package in R software (Subramanian et al., 2005). Pathways with a *p*-value < 0.05 were considered significantly enriched.

### TISIDB database

The Tumor and Immune System Interaction Database (TISIDB) (http://cis.hku.hk/TISIDB) database was utilised to analyse the associations between *PNN* and tumour-infiltrating lymphocytes (TIL), immunosuppressors, and chemokines (Ru et al., 2019).

### Statistical analysis

Statistical analysis was performed using the R software package (version 3.6.1). The differential mRNA expression of *PNN* between tumour tissues and normal controls was compared using Student’s *t*-test. The expression of *PNN* among the clinicopathological parameters groups was compared using Student’s *t*-test and ANOVA. The area under the curve (AUC) of receiver operating characteristic (ROC) was utilised to determine the diagnostic ability of *PNN* and was calculated using the “pROC” R package (Malone et al., 2015). KM curves of disease-free survival (DFS or PFS) of the patients were performed by setting the median expression of *PNN* as the cut-off in the ‘survival’ R package. The log-rank test was used to assess statistical differences, and a cut-off *p*-value < 0.05 was deemed statistically significant.

## Results

### Prognostic and diagnostic value of *PNN* in prostate cancer

**FIGURE 2:**
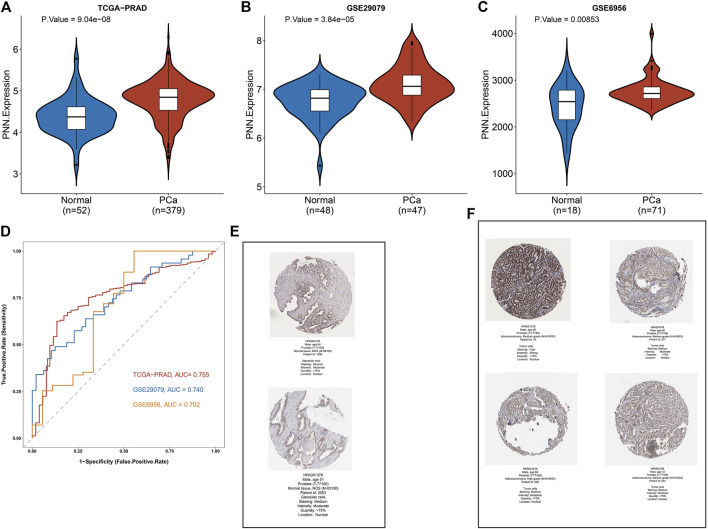
*PNN* expression profile and its diagnostic value in Prostate Cancer (PCa). **(A–C)** Comparison of *PNN* expression levels in the TCGA-PRAD, GSE29079, and GSE6956 datasets. **(D)** The diagnostic value of *PNN* as evaluated by ROC curve. **(E,F)** Immunohistochemistry results of normal (two cases) and PCa tissue (four cases) from the HPA database.

The expression levels of *PNN* between PCa and control samples were compared in the TCGA-PRAD, and the *PNN* expression level was validated with GSE29079 and GSE6956 datasets. As shown in the violin plots, the mRNA expression level of *PNN* was significantly higher in the PCa group in all datasets (Figures 2A–C). Next, we used the same datasets to evaluate the diagnostic value of the *PNN* gene. The accuracy of the diagnostic model was evaluated by ROC curve analysis (Figure 2D). As a result, the AUC of the *PNN* diagnostic model was greater than 0.7 in all three datasets, indicating that the *PNN* gene can be used to discriminate cancer from normal tissues. Moreover, we also observed that the abundance of pinin protein was higher in PCa tissue than in normal tissue (Figures 2E,F).

**FIGURE 3:**
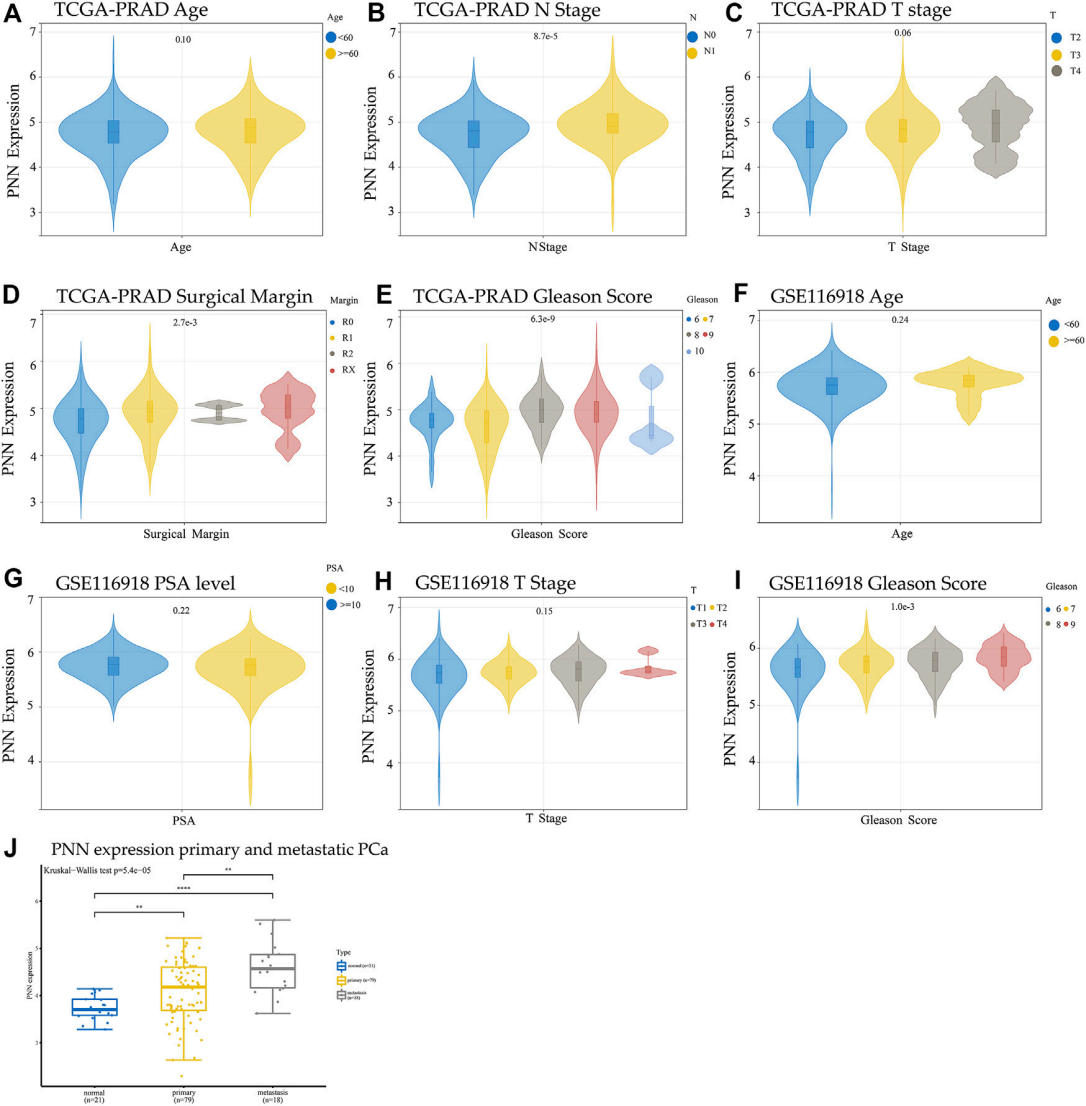
Comparison of *PNN* expression and clinical information in TCGA **(A)** Age, **(B)** N stage, **(C)** T stage, **(D)** Surgical margin, and **(E)** Gleason score. Comparison of *PNN* expression and clinical information of GSE116918 **(F)** Age, **(G)** PSA level, **(H)** T stage, and **(I)** Gleason score. The *t*-test was used to evaluate the difference between two groups, and analysis of variance (ANOVA) was used to compare data divided into more than two groups. **(J)** Comparision of the *PNN* gene expression between primary and metastatic PCa using GSE38241 and GSE25136 datasets following batch effects removal.

To explore the relationship between *PNN* expression and the clinicopathological characteristics in PCa, we compared the *PNN* expression levels according to sample clinical information. The high *PNN* expression was found in the advanced stage of PCa (Figure 3B), and the Gleason scores were strongly correlated with the *PNN* expression levels in PCa patients in both TCGA-PRAD datasets (*p* = $6.3\times 10^{-9}$ ) and GSE116918 dataset (*p* = 0.001) in Figures 3E,I. Collectively, the Gleason score was highly positively correlated with *PNN* expression. Different the surgical margins (R0/1/2/X) found different *PNN* expression (Figure 3D). It has been found that the *PNN* gene expression level was significantly higher in tumors than that of the primary tissue (Figure 3J, data process in Supplementary Figure S1), suggesting this gene can be used for diagnostics in metastatic patients. Age (Figures 3A,F), T stage (Figures 3C,H), or PSA level (Figure 3G) are not correlated with the *PNN* expression’s significance.

**FIGURE 4:**
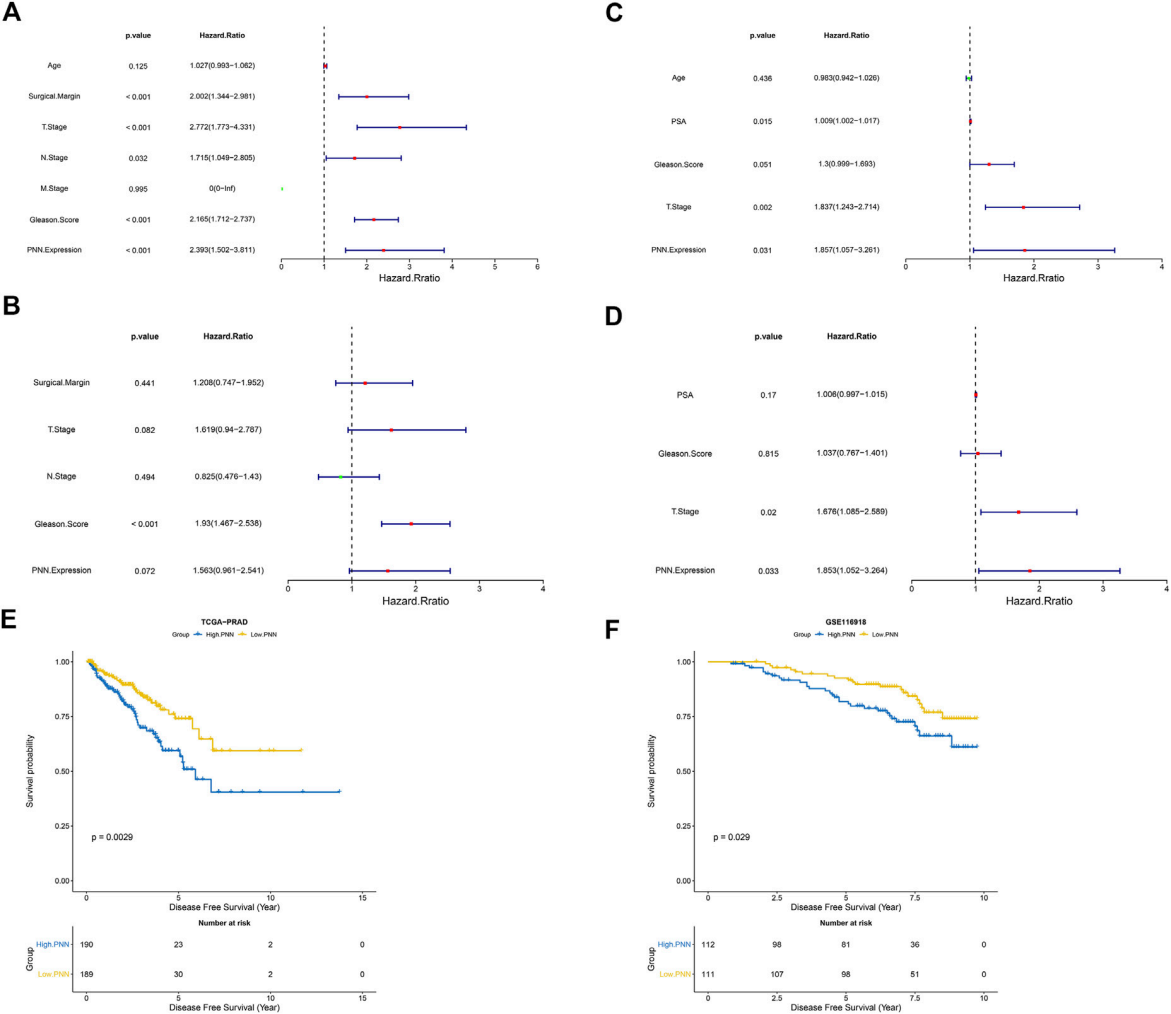
*PNN* prognostic value in the TCGA-PRAD and the GSE116918 cohorts. Forest plots of univariate and multivariate Cox regression analysis for the TCGA cohort **(A)** univariate, **(B)** multivariate and the GSE116918 cohort **(C)** univariate, **(D)** multivariate. **(E,F)** DFS curves plotted according to the KM method for the TCGA-PRAD and GSE116918 cohorts using the log-rank test.

Univariate and multivariate Cox analyses were conducted to investigate the independent prognostic factors in TCGA-PRAD and validated with GSE116918 datasets. The univariate analysis in the TCGA-PRAD dataset indicated that the surgical margin, T stage, N stage, Gleason score, and *PNN* expression were associated with the prognosis of PCa patients (Figure 4A). In contrast, multivariate Cox regression analyses in the same dataset demonstrated that only the Gleason score could be used independently to predict the prognosis of patients **(** Figure 4B). Similarly, the PSA levels, Gleason score, T stage, and *PNN* expression were found to be significant risk factors by univariate Cox analysis in the GSE116918 dataset (Figure 4C). In the same dataset, multivariate Cox regression analyses demonstrated that T stage and *PNN* expression could be used independently to predict the prognosis of patients (Figure 4D). We then validated these findings by analysing the DFS curves of the *PNN* high- and low-expression groups, which showed that the *PNN* high-expression group had remarkably worse survival rates than the low-expression group in both the TCGA-PRAD and the GSE116918 datasets (Figures 4E,F). The hazard ratio of *PNN* was greater than 1 in both datasets. Taken together, it suggested that *PNN* was a risk factor in the prognosis of PCa. However, the independent prognostic value of *PNN* needed further investigation and confirmation.

### *PNN* co-expression network and potential drug targets in prostate cancer

To identify pharmaceutical molecules with DsigDB database and further uncover the biological processes *PNN* participated, the co-expression pattern of *PNN* in PCa was explored. All co-expressed genes are listed in Supplementary Table S2.

**FIGURE 5:**
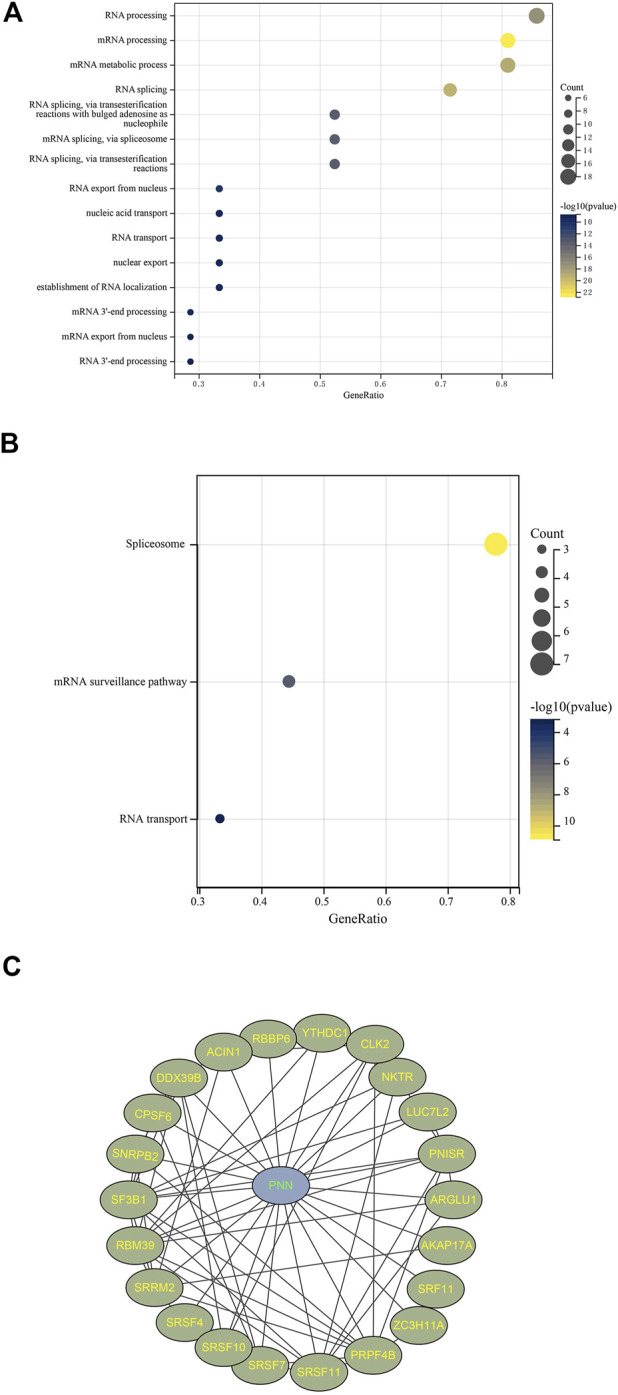
Co-expressed network with *PNN*. Enrichment results filtered with FDR < 0.05 based on 25 uniquely interacted and co-expressed genes with *PNN* with **(A)** GO and **(B)** KEGG. **(C)** Protein-Protein-interaction (PPI) network constructed using Cytoscape 3.8.2 based on *PNN* and 25UC.

BioGrid hosted 243 proteins interacting with pinin extracted from published literature. A total of 368 genes were co-expressed with pinin following the criteria of r > 0.6 and *p* < 0.05, of them, twenty-five genes overlapped with 243 interactive proteins of pinin (25UC for short). Those 25UC genes were enriched in RNA splicing and RNA/mRNA processing based on GO enriched analysis (Figure 5A) and enriched in the spliceosome, mRNA surveillance pathway, and RNA transport based on KEGG enrichment analysis (Figure 5B). These results suggest that *PNN* is mainly linked to the RNA process and RNA transport in PCa. *PNISR*, *RBM39*, *DDX39B*, *SF3B1*, *SRSF11*, *CPSF6*, *CLK2*, and *SNRPB2* have the function of splicing or process of RNA; *ACIN1* and *NKTR* participate in cell apoptosis and immune response. The protein-protein interaction network can be found in Figure 5C.

To explore the potential therapeutic targets in PCa, we focused on those genes that strongly positively (*r* > 0.8 and *p* < 0.05) correlated with upregulated *PNN*, including *FNBP4*, *TCERG1*, *RBM39*, *DDX39B* and *DMTF1*. Ten possible pharmaceutical molecules were identified using the Enrichr package from the DsigDB database, based on their *p*-value. Table 1 lists the effective drugs from the DsigDB database for PCa.

**TABLE 1:** List of the suggested drugs for PCa patients with *PNN* expression.

Drug	*p*-value	Drug indication	Drug stage (approved or not)	Targeted gene	References
Valproic acid	7.43E-06	To control complex partial seizures and both simple and complex absence seizures	FDA approved	HDAC9	Kanai et al. (2004)
Vorinostat	1.19E-05	The treatment of cutaneous manifestations in patients with progressive, persistent, or recurrent cutaneous T- cell lymphoma (CTCL) following prior systemic therapies	Phase III for the treatment of cutaneous T cell lymphoma (CTCL),Mesotheliomas, Multiple Myeloma (MM)	HDAC1,HDAC2, HDAC3, HDAC6	Chen et al. (2002), Xu et al. (2007)
Cephaeline	1.42E-05		Experimental		
Fisetin	1.74E-05		Experimental	CDK6	Berman et al. (2000)
Trichostatin A	1.75E-05		Phase I: Relapsed or refractory hematologic malignancies	HDAC7	Berman et al. (2000), Komata et al. (2005)
				CASP8	
Glibenclamide	2.45E-05	Diabetes mellitus type II	FDA approved	Q09428	Serrano-Martín et al. (2006), Ueda et al. (1999)
				Q14654	
Vitamin E	4.50E-05	Vitamin deficiency	Being considered safe by the FDA	SEC14L4	Schmölz et al. (2016)
Camptothecin	4.54E-05		Experimental	TOP1	Chen et al. (2002)
0175029-0000	1.27E-04		Experimental		
Doxorubicin	2.10E-04	various cancers and Kaposi’s Sarcoma	FDA approved	TOP2A	Menendez et al. (2006)

### DNA methylation concerning *PNN*

**FIGURE 6:**
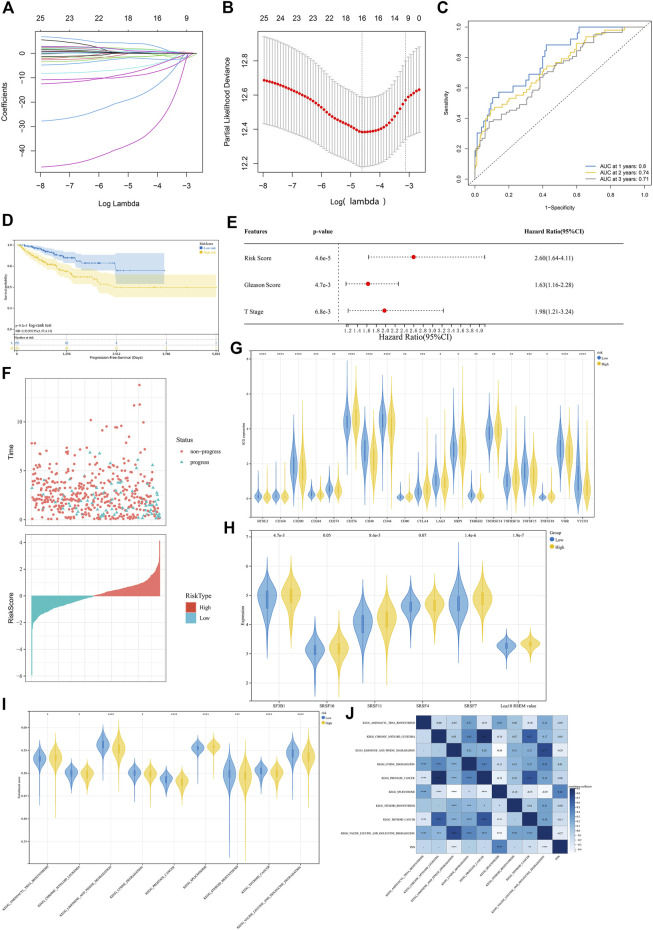
Analysis of DNA methylation levels concerning the *PNN* expression. **(A)** LASSO model tuning parameters ( λ , lambda) were selected by 10-fold cross-validation. **(B)** LASSO coefficient profiles of 180 CpG sites. **(C)** ROC Curves of the risk model performed in the TCGA-PRAD cohort’s CpG sites methylation data. ROC, receiver operating characteristics. **(D)** Kaplan Meier (KM) plot for a discriminative median patient risk score with two methylation sites about PFS. **(E)** Hazard ratio and *p*-value of constituents involved in multivariate Cox regression and some parameters of the CpG-site signature. **(F)** The distribution of the PFS status of patients in the high-risk and low-risk groups. **(G)** Different levels of each immune checkpoint (Danilova et al., 2019) between high and low-risk groups using violin plots and the Wilcoxon test. *p < 0.05; **p < 0.01; ***p < 0.001; ****p < 0.0001. **(H)** Comparison of splicing factors in 25UC gene set that retrieved from Biogrid and splicing variants expression between high and low-risk groups following ssGSEA by the Wilcoxon test. Log10 RSEM value, Expression of *PNN* splicing isoforms. **(I)** Comparison of enrichment score (ES) > 0.5 between high and low-risk groups following ssGSEA by the Wilcoxon test. Only significant difference is displayed. *p < 0.05; **p < 0.01; ***p < 0.001; ****p < 0.0001. **(J)** *PNN* expression level correlation with the enrichment scores by Spearman test.

After excluding missing values, a total of 180 CpG sites in the *PNN* and its co-expressed genes *FNBP4*, *TCERG1*, *RBM39*, *DDX39B* and *DMTF1* (r > 0.8 and *p* < 0.05) promoter regions were extracted from TCGA-PRAD methylation data. Univariate Cox regression analysis showed that 25 CpG sites were significantly correlated with PFS. Following the LASSO algorithm, 16 CpG sites were selected (lambda = 0.009914324, Figures 6A,B). A model was then constructed with multivariate Cox regression. We constructed a risk score system Eq. 1 with seven CpG sites.

Risk Score=−17.451×cg04787786−2.182×cg09878914+3.097×cg16316344−38.055×cg16408528+1.652×cg17114847−46.828×cg17439097+1.943×cg25800328 (1)

Eq. 1

The areas under the ROC curves (AUC) of 1-, 2-, and 3-year PFS were 0.80, 0.74 and 0.71, respectively (Figure 6C), indicating the good performance of the risk score signature. We noticed that this risk score was linked to the PFS status of the PCa patients (Figure 6D), indicating that this risk score could be used to predict the progression of PCa. Multivariate Cox regression confirmed that the risk scores could also be an independent prognostic factor (Figures 6E,F). In addition, the expression level of 47 immune checkpoint genes (ICG) proposed by Danilova et al. (2019) were compared between high and low-risk groups with the Wilcoxon test based on the signature constructed above. As a result, *CTLA4*, *CD276*, *CD80*, *NRP1*, *TNFRSF18*, *TNFSF18* and *TNFRSF14* were found to be significantly higher in the high-risk group, while the expression levels of *BTNL2*, *CD160*, *CD200*, *CD244*, *CD274*, *CD40*, *CD44*, *LAG3*, *TMIGD2*, *TNFSF15*, *VSIR* and *VTCN1* were reduced significantly in the high-risk group (Figure 6G). Then, a ssGSEA was performed using the KEGG database to explore different molecular mechanisms between the high- and low-risk groups. Among significantly enriched pathways (*p* < 0.05), the top 10 were compared between high and low-risk groups. Between the two risk groups, the splicing factors genes, such as *SF3B1*, *SRSF11* and *SRSF7*, are significantly higher in high risk group based on the median (Figure 6H). The expression of *SRSF10* (*p* = 0.05) and *SRSF4* (*p* = 0.07) were marginally higher. Moreover, the splicing isoforms expression was also significantly increased in the highly risky group based on the median. Besides prostate cancer and other cancer pathways, the risk model also found significant different enrichment scores in spliceosome and biogenesis and degradation pathways (Figure 6I). The correlation between the pathways and the *PNN* expression is illustrated in Figure 6J.

### Immune infiltration

**FIGURE 7:**
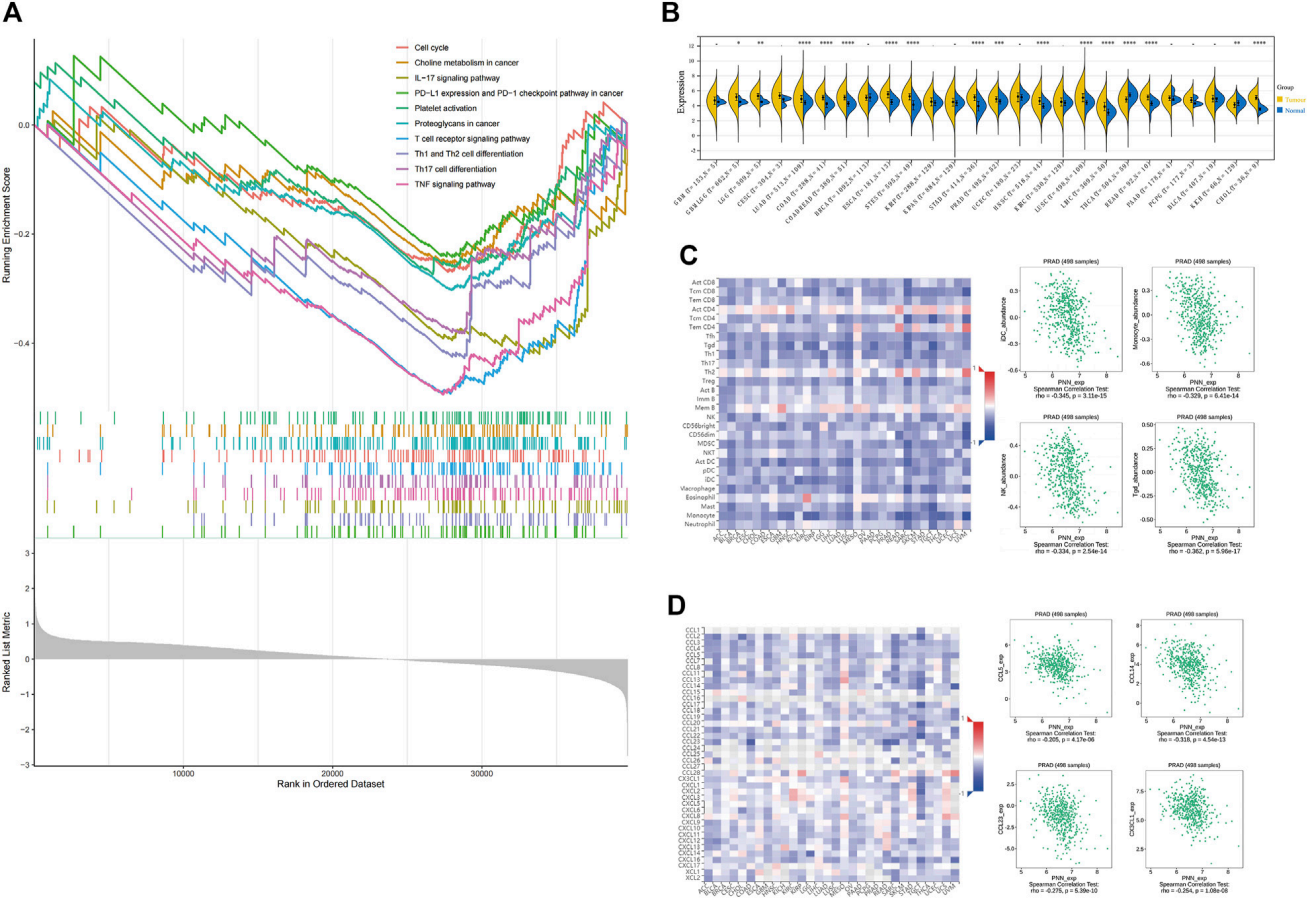
Immune infiltration. **(A)** GSEA results are based on *PNN* high- and low-expression groups. **(B)** *PNN* expression profile in pan-cancers. Unpaired Wilcoxon Rank Sum and Signed Rank Tests for significance of differences analysis. Analysis of the correlation between *PNN* expression and **(C)** tumor-infiltrating lymphocytes (TIL) and **(D)** chemokines. The left figure shows the correlation between *PNN* expression in TCGA pan-cancer and TIL, immunosuppressants, and chemokines, and the Scatter plot (right) clearly shows correlations with *PNN* expression in PCa patients.

To infer the pathways by which *PNN* genes were involved in the development of PCa, GSEA enrichment was performed on the *PNN* high- and low-expression groups. Among the enriched pathways (adjusted *p* < 0.05) (Supplementary Table S1), we noted that immune-related pathways were enriched, including the IL-17 signalling pathway, the T cell receptor signalling pathway, Th1 and Th2 cell differentiation, Th17 cell differentiation, and the TNF signalling pathway. In addition, cancer-related pathways, such as the cell cycle, choline metabolism in cancer, PD-L1 expression and PD-1 checkpoint pathway in cancer, and proteoglycans in cancer were also enriched in PCa (Figure 7A). We calculated the expression difference between normal and tumour samples in each tumour, and observed significant upregulation in 14 tumours (Figure 7B). Subsequently, the correlation between *PNN* and immune infiltration was executed to broaden the cognition of the correlation between *PNN* and TIL, immune inhibitors, and chemokines in PCa. As to TIL, *PNN* expression was negatively correlated with iDC, monocyte, NK cell, and Tgd in Figure 7C (rho < −0.3 and *p* < 0.05). Figure 7D showed the correlations between *PNN* expression and chemokines, of which CCL14 was negatively correlated with *PNN* (*r* < −0.3 and *p* < 0.05).

## Discussion

Prostate cancer remains one of the most common cancers, with a poor patient prognosis upon transition to metastasis (Phillips et al., 2020). It is urgent to continually identify proteins or hormones related to this disease for potential novel treatments or as potential biomarkers for early detection. It has been suggested that *PNN* promotes the epithelial-mesenchymal transition in tumours, which is the preliminary stage of metastasis (Vandamme et al., 2020; Dai et al., 2021; Zhang et al., 2022). The gene encodes a transcriptional activator binding to the E-box 1 core sequence of the E-cadherin promoter and upregulates E-cadherin expression implicated in tumour metastasis as a promoter of growth and metastasis (Na et al., 2020). In this study, we comprehensively analysed TCGA and GEO PCa datasets with bioinformatics approaches, which provided relations of *PNN* with PCa on the molecular pathway level. Similarly, these results indicated that the *PNN* gene could be a useful prognosis feature for PCa patients in clinical practice related to tumour progression and poor prognosis (Wei et al., 2016; Yang et al., 2016; Tang et al., 2020; Jin et al., 2021).

We identified several FDA-approved drugs potentially useful for PCa patients. Valproic acid (VPA) is a neuroprotective agent used for disease relating to neurological diseases (Kale et al., 2021). It is reported that through autophagy and apoptosis, VPA synergistically enhances anticancer effects with Arsenic trioxide in lung cancer cells (Park et al., 2020) and advanced patients in clinical Phase I (Atmaca et al., 2007). Another identified drug is Vorinostat which is applied to cutaneous T cell lymphoma (Dumont et al., 2014). In fact, some research has applied this drug to PCa patients as a sensitiser in therapy for PCa (Stiborova et al., 2012; Jonsson et al., 2016). Trichostatin A comes from the same family as Vorinostat; it inhibits histone deacetylases (*HDAC*) and is a broad spectral drug for various cancers. Additionally, the natural products and anti-proliferative agents, Camptothecin (and its derivative) (Zhang et al., 2000; Chiu et al., 2020) and Fisetin (Kashyap et al., 2019), are able to induce apoptosis and interfere with the cell cycle. Cephaeline inhibits cancer cells’ growth, migration and proliferation (Silva et al., 2021). The progression of the PCa tumours can be hindered by the medication of those drugs or molecules. The drug sensitivity data were integrated to identify those drugs with variant sensitivity in different subgroups. By proposing promising therapy candidates for targeted treatment for PCa patients, our results provide an additional selection of the clinical practice for treating PCa patients. By contrast, due to the ambiguous role of Vitamin E (Yang et al., 2020) and Glibenclamide potentially causing death (Monami et al., 2006), these two drugs are not suitable for clinical application. Doxorubicin, a highly effective anticancer drug, induces many cardiotoxic effects (Ferreira et al., 2019); hence, it is not recommended so far.

Several studies elucidate the critical role of RNA splicing in cancer pathogenesis (Inoue et al., 2019; Shuai et al., 2019; Suzuki et al., 2019; Wang et al., 2020). With GO and KEGG enrichment analysis, we found that the gene *PNN*, together with its 25UC gene set, is involved in RNA splicing. We could conclude that *PNN* might play a role in RNA splicing by participating in spliceosomes. It has been suggested that tumour pathogenesis is influenced by splicing resulting from abnormal splicing that is widespread in cancer, such as dysregulation of splicing and aberrant splicing patterns (Ryan et al., 2015; Seiler et al., 2018; Wang et al., 2020). Thus, we believe that dysfunction of the *PNN* gene will affect the normal function of the spliceosome, which will result in many aberrant mRNAs because of abnormal splicing.

The GSEA based on *PNN* high- and low-expression groups offered possible pathways related to immune infiltration. Nevertheless, we observed that plenty of genes were co-expressed with *PNN* gene; hence, it is hard to state that *PNN* was related to immune infiltration. The tumours with increased *PNN* expression shared a similar correlation pattern with TIL and chemokines. Thus, *PNN* could be a indictor for TIL. We also performed ssGSEA to explore the potential mechanism of *PNN* in RNA splicing with the high- and low-risk groups of PCa. Among all the differently enriched pathways, it has been found that the high-risk group showed higher enriched scores in the spliceosome pathway and *PNN* expression also positively correlated with this pathway. The hypomethylation status of CpG sites in the *PNN* gene promoter probably resulted in an increased *PNN* expression and then potentially contributes to the progression of PCa. The significantly increased expression of several splicing factor genes, such as *SF3B1*, *SRSF11* and *SRSF7* (Figure 6H), in the high-risk group suggested that abnormal splicing was associated with an increased risk for PCa, such as progression.

We attempted to comprehensively determine the potential underlying mechanisms of *PNN* on PCa progression. Therefore, we also explored the role of epigenetic markers in PCa. DNA methylation is an epigenetic marker that is essential in regulating gene expression. DNA methylation of CGIs is essential for gene expression and tissue-specific processes. Previous reports indicate that DNA methylation at promoters negatively correlates with gene expression (Chen et al., 2016; Keller et al., 2016; Neri et al., 2017). Aberrant methylation of *PNN* CGIs was correlated with changed *PNN* expression (Akin et al., 2016). Using the methylation status of CpG sites in the *PNN* gene and its co-expressed genes, we constructed a prognostic signature. This signature suggested that the methylation status of CpG sites may play a role in the prognostic prediction, while the combined methylation signature might provide better potential for achieving more sensitive and specific prognostic value in PCa patients. The prognostic value of these DNA methylation signatures has not been intensively explored yet. We have found that the high- and low-risk groups could respond differently to the immune therapies, suggesting the classification of PCa is meaningful (Xia et al., 2021). Dividing patients based on their risk scores would be a direction of precision therapeutics, which will be facilitated by classifying PCa (Wu et al., 2021). Therefore, the present study provides a new insight that a combination of epigenetic biomarkers may improve risk stratification and survival prediction in PCa patients.

Based on those results, the pinin protein should participate in the biological activity of spliceosomes or splicing (Kim et al., 2017). Pinin is an exon junction component (EJC), which is a member of the spliceosome complexes (Akin et al., 2016). Although the mechanism underlying *PNN* promoting tumourigenesis is rarely reported, *SF3B1*, a well-known spliceosome-associated gene and co-expressed with *PNN* (*r* > 0.8), is linked to a variety of solid tumours, including PCa (Rahman et al., 2020; Yang et al., 2022). Therefore, as a member of the spliceosome, mutations in *PNN* could rewire its interactions with other proteins in the spliceosome, including the *SF3B1* gene, which will lead to splicesome dysfunction, and enhance the activation of the NF- ĸ B pathway (Pollyea et al., 2019; Yang et al., 2022).

Through bioinformatics analyses, we have explored the differential expression pattern of *PNN* between normal and PCa patients, its independent prognostic value, the potential regulatory mechanisms, the relationship with immune infiltration, and the co-expression genes. We validated our results to prove our results using external datasets. Since our results solely come from data analysis, experimental verification will need to support further the rationale of the molecular mechanisms underlying PCa progression. In conclusion, *PNN* is a potentially valuable biomarker for PCa diagnosis and patient management. Furthermore, we have identified the potential new drugs as well as the ICGs that could be utilised in immune therapy for PCa treatment for PCa patients with high expression of *PNN*.

## Data Availability

The original contributions presented in the study are included in the article/Supplementary Material, further inquiries can be directed to the corresponding authors.
