# Supplementary material for: Prognostic value of PNN in prostate cancer and its correlation with therapeutic significance
Source: Front Genet. 2022 Nov 16;13:1056224. doi: 10.3389/fgene.2022.1056224 (PMC9708726; doi:10.3389/fgene.2022.1056224)
Supplement: Supplementary file 3 [file DataSheet1.docx]

Supplementary Material

# Batch effects removal for expression profiles of *PNN* gene in primary and metastatic PCa

For the batch effects removal for expression profiles of *PNN* gene in primary and metastatic PCa, the multiple independent datasets from GEO (GSE38241 and GSE25136) were utilized for the purpose. From the box plot (**Supplementary Figure 1**a, c &e), it is obvious there were batch effects. Following batch effects removal by COMBAT, the batch effects have been removed (**Supplementary Figure 1**b, d &f).


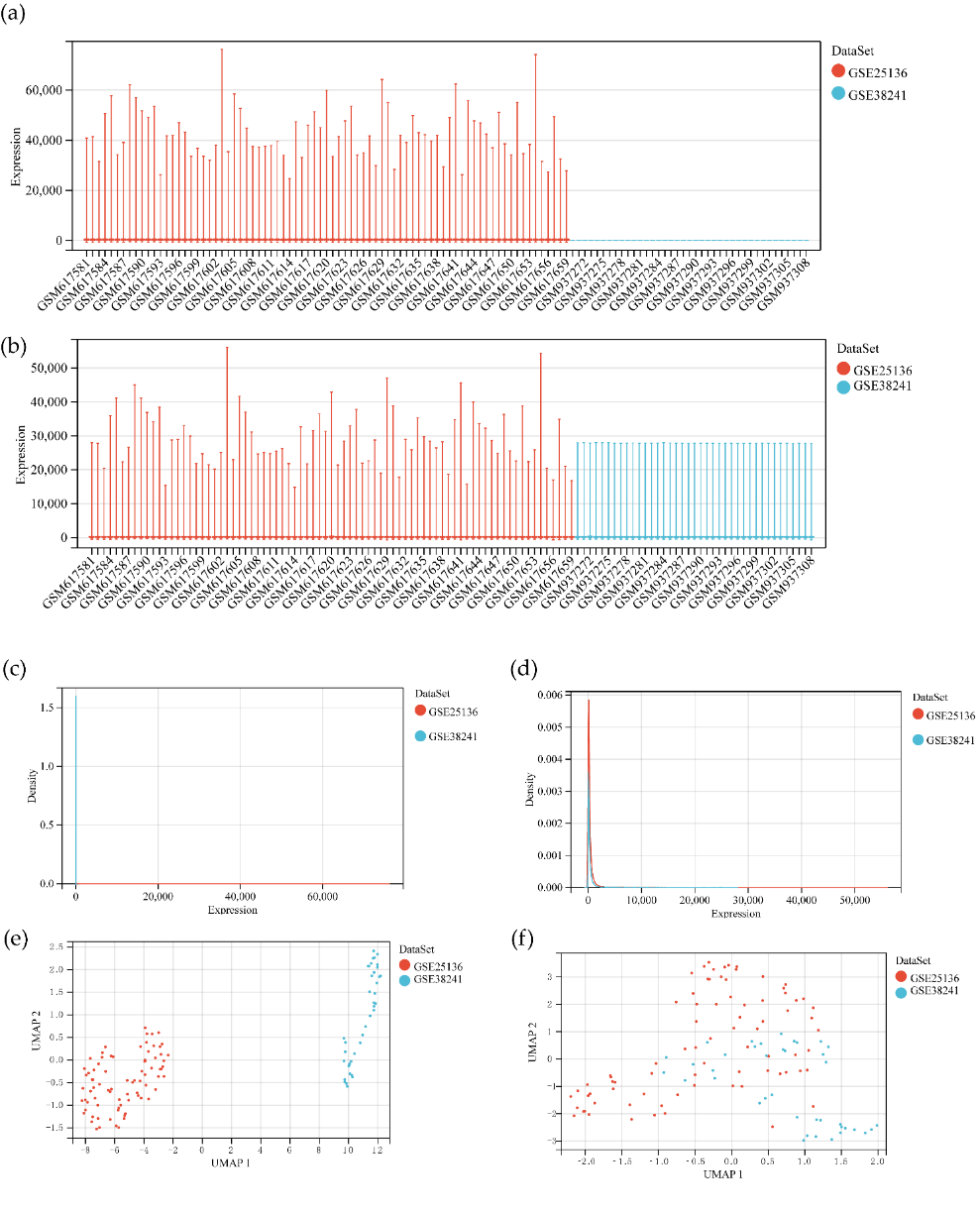


**Supplementary Figure 1.** Batch effect removal for expression profiles of *PNN* gene. (a) Boxplot of 2 datasets (a) before and (b) after batch effect removal. Density plot of 2 datasets (c) before and (d) after batch effect removal. The UMAP plot (e) before and (f) after batch effect removal.
